# Artificial intelligence-based forensic sex determination of East Asian cadavers from skull morphology

**DOI:** 10.1038/s41598-023-48363-3

**Published:** 2023-11-29

**Authors:** Hiroki Kondou, Rina Morohashi, Satoko Kimura, Nozomi Idota, Ryota Matsunari, Hiroaki Ichioka, Risa Bandou, Masataka Kawamoto, Deng Ting, Hiroshi Ikegaya

**Affiliations:** Department of Forensic Medicine, Graduate School of Medicine, Kyoto Prefectural University of Medicine, 465 Kajiicho, Kawaramachi-Dori Hirokoji-Agaru, Kamigyo-Ku, Kyoto, 602-8566 Japan

**Keywords:** Computational biology and bioinformatics, Anatomy

## Abstract

Identification of unknown cadavers is an important task for forensic scientists. Forensic scientists attempt to identify skeletal remains based on factors including age, sex, and dental treatment remains. Forensic scientists commonly consider skull or pelvic shape to evaluate the sex; however, these evaluations require sufficient experience and knowledge and lack objectivity and reproducibility. To ensure objectivity and reproducibility for sex evaluation, we applied a gated attention-based multiple-instance learning model to three-dimensional (3D) skull images reconstructed from postmortem head computed tomography scans. We preprocessed the images, trained with 864 training data, validated the model with 124 validation data, and evaluated the performance of our model in terms of accuracy with 246 test data. Furthermore, three forensic scientists evaluated the 3D skull images, and their performances were compared with those of the model. Our model showed an accuracy of 0.93, which was higher than that of the forensic scientists. Our model primarily focused on the entire skull owing to visualization but focused less on the areas often investigated by forensic scientists. In summary, our model may serve as a supportive tool to identify cadaver sex based on skull shape. Further studies are required to improve the model’s performance.

## Introduction

Forensic identification of cadavers may be challenging in cases of skeletal remains or advanced putrefied or burned cadavers^1^. However, the sex may still be identified in these cases owing to the differences between male and female bone morphology. For example, the male skull is rugged and has a square mandible, whereas the female skull is smoother and has a rounded mandible. In addition, the male temporal ridge is more prominent than that of the female^2,3^. Furthermore, the male pelvic inlet is heart-shaped, whereas that of the female is oval-shaped. The male pelvis itself is heavier and thicker than that of the female^4,5^. Furthermore, the male epicondylar breadth of the femur is greater than that of the female^6^. As described above, several differences in bone morphology between the sexes are well known.

**Figure 1:**
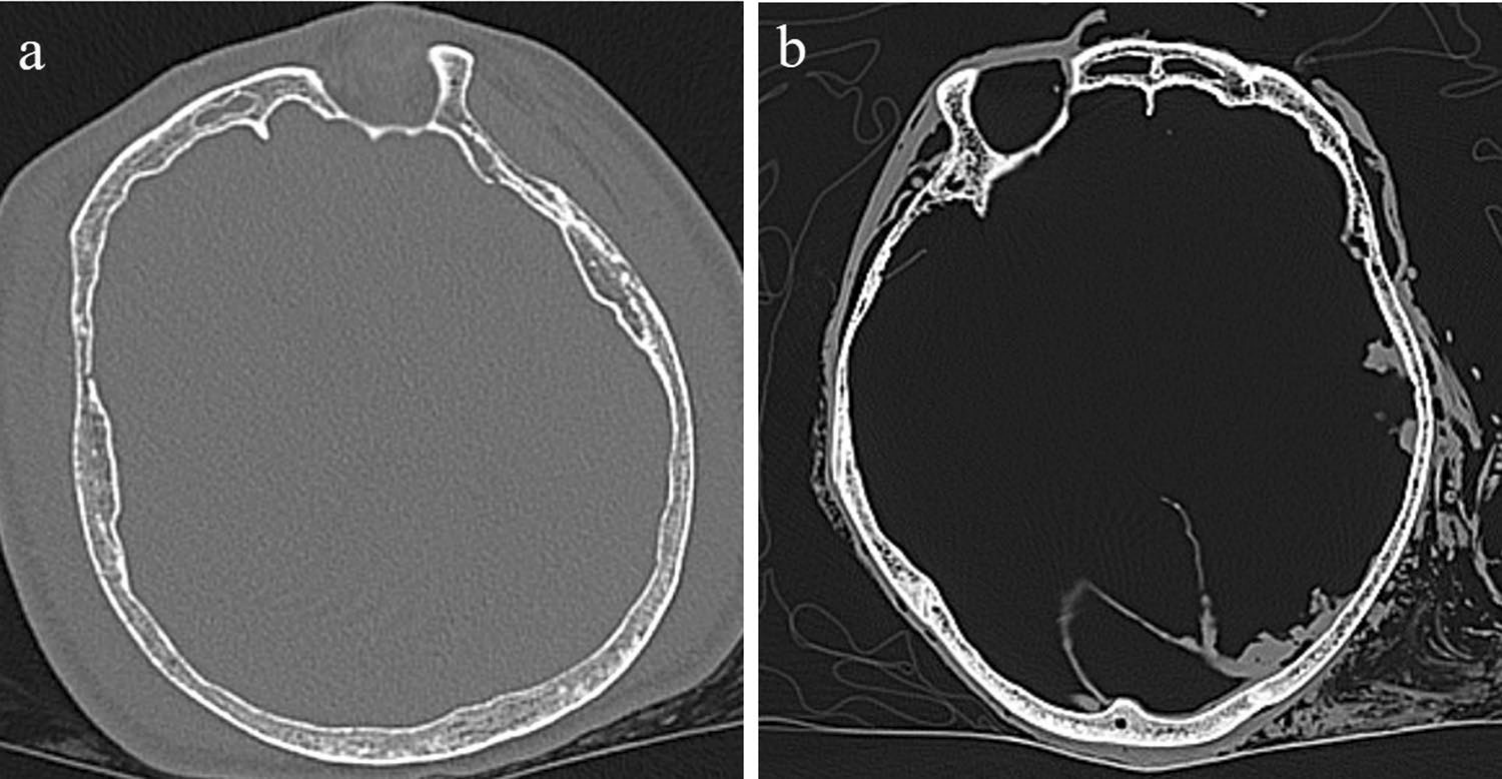
Comparison between the short and long postmortem intervals. (**a**) The CT slice within 12 h from the time of death. (**b**) The CT slice more than 1 month after the time of death. The putrefied gas can be confirmed in the cancellous bone of the skull.

Evaluation of bone morphology requires sophisticated knowledge and sufficient experience. Therefore, experienced forensic scientists or forensic anthropologists generally perform this evaluation. However, this evaluation is subjective and may lack reproducibility. Recently, the global advancement of artificial intelligence has resulted in its wide applications in several medical fields^7–9^, including in forensic medicine. Several studies have used machine-learning algorithms, a field of artificial intelligence, to evaluate the sex from skull shape and achieved high accuracies in forensic sex determination. We found two approaches in the literature: directly evaluating skull shape^10^ or using Computed Tomography (CT)^11–15^. We suggest that the method using CT is superior to a direct evaluation in terms of convenience. For example, methods of direct evaluation are not applicable in non-skeletonized cases, such as burned bodies or adipocere, which are also targets of identification by forensic experts. Regarding the CT methods, most studies used CT images as training datasets obtained from living individuals including volunteers and patients. However, whether the machine-learning models trained with data from living people can be applied to unknown cadavers remains questionable owing to the multiple differences between living and dead people. First, most cadavers undergo putrefaction, which typically results in the generation of putrefied gas throughout the entire body depending on the degree of putrefaction (Fig. 1). This gas could cause a partial volume effect on CT images visually stronger than the images with no gas. This phenomenon can occur when several substances are mixed in one voxel. The Hounsfield Unit (HU) values of a voxel indicate the average HU values of the substances within that voxel, which may result in the blurring of the boundaries or misestimation of density. Skull CT images of living people whose skulls are surrounded by soft tissue including brain or subcutaneous tissue may differ from skull CT images of putrefied cadavers whose skulls are surrounded by soft tissue and putrefied gas, according to the above mentioned phenomenon. In other words, HU values of the outer edge or outer line of the skull represent the mixed HU values of the soft tissue and skull in living people. HU values of putrefied cases represent a mixture of air and the skull. Therefore, the HU value of voxels of the outer line of the skull from putrefied cases could be lower compared to those of live individuals. This decrease in HU values may cause subtle changes to the entire skull shape. Furthermore, CT images of partially defective skulls are rarely obtained from volunteers or hospitalized patients. Identification of partially defective skulls may be a task for forensic scientists. A previous study of postmortem CT scans^13^ evaluated the whole skull, cranium, and mandible. However, we considered that machine-learning models should be trained with postmortem CT scans that contain putrefied or defective cases because forensic scientists are additionally tasked with the identification of such cases. Our main purpose was to apply the machine-learning models for forensic cases such as putrefied cases and achieve higher performance than the performance of specialized forensic scientists. Besides, we also prioritized the presentation of the explainability and interpretability of machine-learning models because most forensic scientists do not have enough knowledge about machine-learning to interpret the results. To the best of our knowledge, there are no published reports utilizing deep learning to investigate the interpretation of important regions of human cadaver. Depicting and interpreting the important regions for sex classification would contribute not only to forensic medicine but also to forensic anthropology.

## Results

### Cadaver characteristics

**Figure 2:**
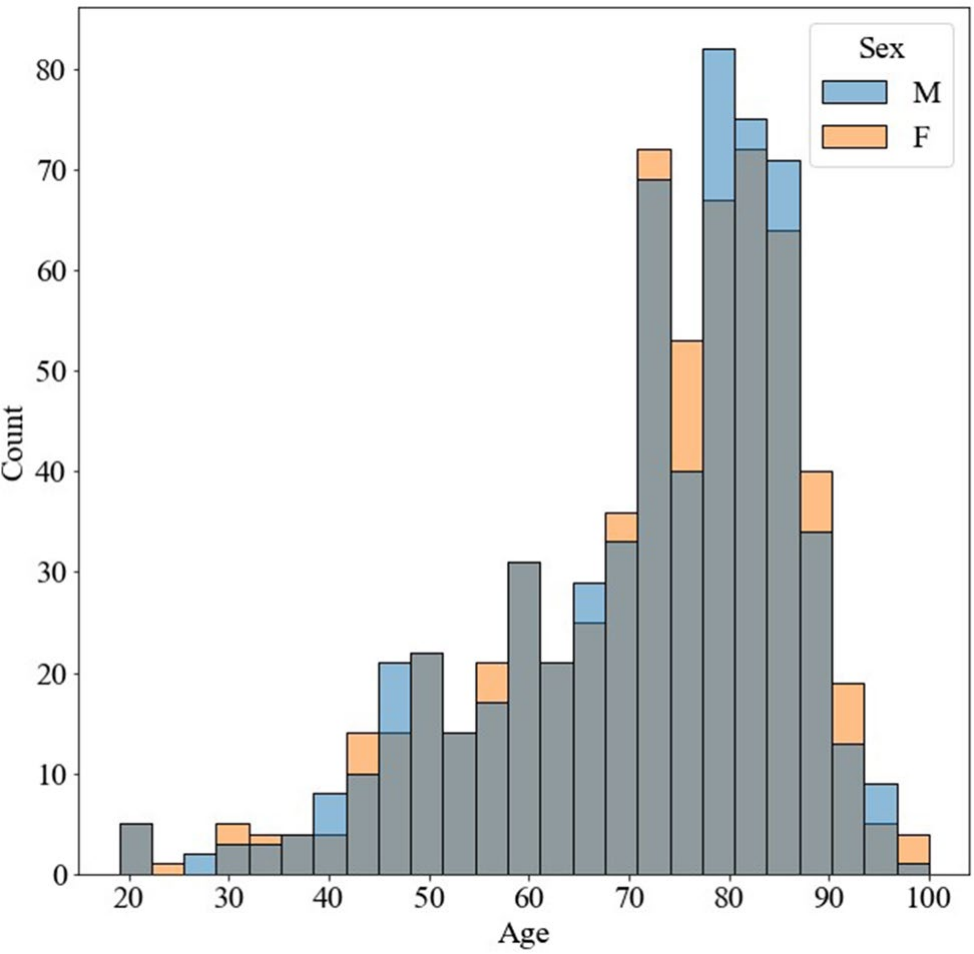
Age histogram of each sex. M and F indicate male and female, respectively. The blue bar depicts the male and the orange the female. Overlapped regions are grey.

We performed a postmortem CT examination for 2041 cadavers (1397 males and 644 females). We performed 1:1 propensity score matching, and 1234 cadavers were selected and randomly divided into 864 training data, 124 validation data, and 246 test data. The median ages with interquatile ranges (IQRs) of males and females were 76 (64–82) years and 75 (64–82.5) years old, respectively. Figure 2 shows the age distribution.

The postmortem interval (PMI) was approximately 8 h to 7 months for males and approximately 8 h to 8 months for females. In this study, PMI was defined as the duration from the estimated time of death to postmortem CT examination. We could not estimate the PMI for cases with insufficient information; therefore, these ranges do not show the results of all the cadavers. Some cases had a PMI over 1 year, and those cases did not have clues to accurately estimate the PMI.

### Model parameters

The optimizer and hyperparameters are shown in Table 1. Other parameters not described in Table 1 were default settings. Deep-learning models are generally trained to minimize the loss function, which is the difference between the true value and the predicted value. Optimizers, such as AdamW, are algorithms used to calculate the loss function minimum. Hyperparameters are external configuration variables used by researchers to manage the training of deep-learning models. The learning rate is a fixed number that determines the magnitude at which the optimizer updates parameters. Weight decay is commonly set to avoid overfitting. Overfitting is a state where models present high accuracy for training datasets but low accuracy for test datasets. Dropout is also a method used to avoid overfitting by deactivating some degrees of Neural Network nodes. Epochs indicate the number of training, and Batch size determines the number of samples used in one iteration.

**Table 1:** Optimization details.

Optimizer	Learning rate	Weight decay	Dropout rate	Epochs	Batch size
AdamW	0.0001	0.0005	0.3	1000	1

### Sex prediction results

**Figure 3:**
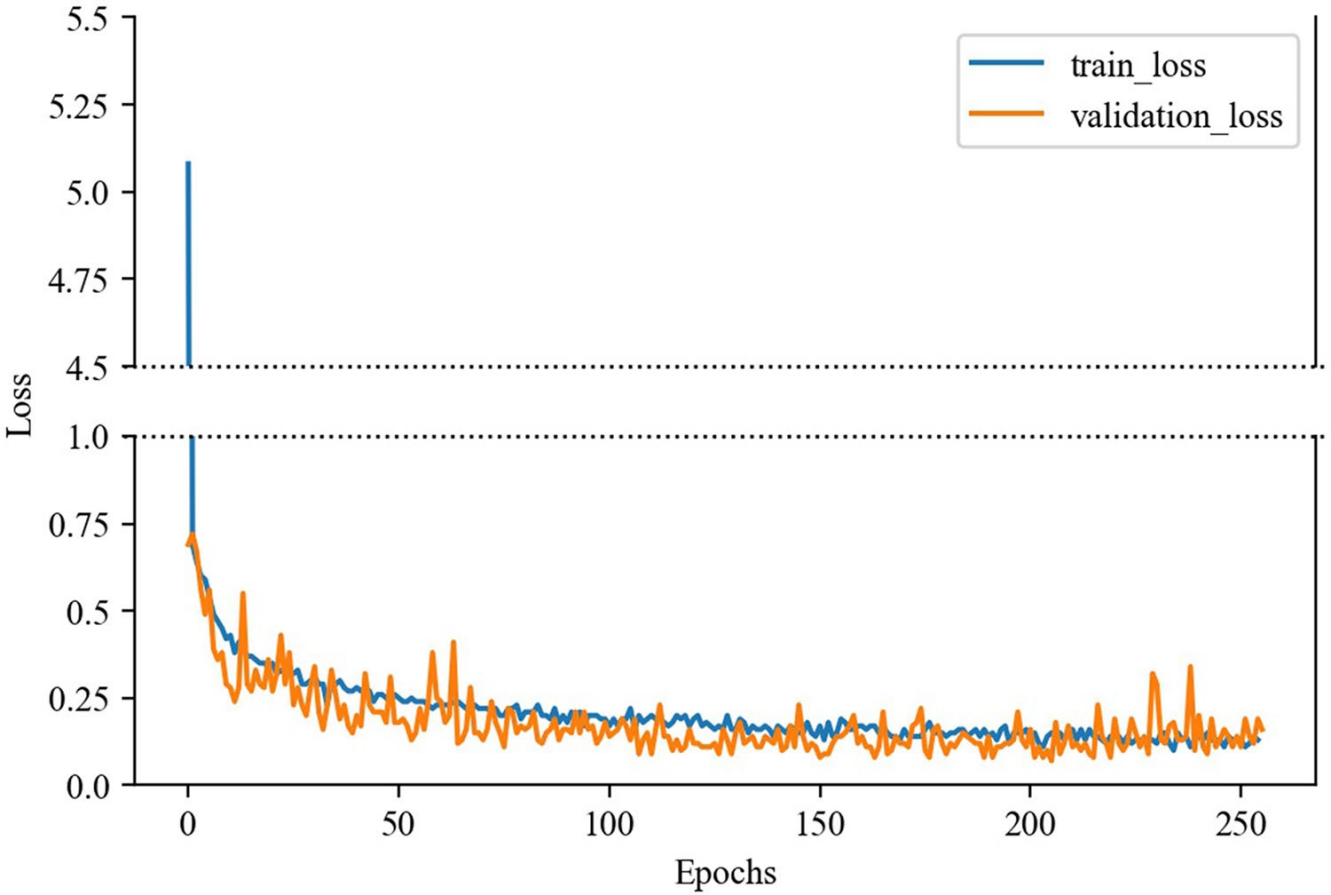
Loss curve of the training. The blue and orange lines show the training loss and validation loss, respectively.

The training procedure stopped at 254 epochs by the “EarlyStopping”. The loss curve is depicted in Fig. 3. The accuracies for the training, validation, and test dataset were 0.94, 0.96, and 0.93, respectively. The sensitivity and specificity for females were 0.92 and 0.95, respectively. The multiple-instance Learning (MIL) model results for the test dataset are shown in Table 2.

**Table 2:** The confusion matrix of our MIL model.

		Prediction		Total
		Female	Male	
True	Female	113	10	123
	Male	6	117	123
		119	127	246

The comparison between the MIL model and three forensic scientists is shown in Table 3. The 95% confidence intervals for the difference in accuracy between the MIL model and each evaluator are shown in Table 4. Our MIL model presented higher accuracy than the trained forensic scientists.

**Table 3:** The comparison of accuracy and sensitivity and specificity for females for the test dataset between the MIL model and humans.

	MIL model	Trained A	Trained B	Beginner
Accuracy	0.93	0.83	0.77	0.63
Sensitivity	0.92	0.88	0.64	0.61
Specificity	0.95	0.79	0.89	0.65

**Table 4:** The 95% confidence intervals of accuracy and sensitivity and specificity for females between the MIL model and humans.

	Trained A	Trained B	Beginner
Confidence interval of accuracy	0.05–0.16	0.11–0.23	0.24–0.37
Confidence interval of sensitivity	− 0.035–0.12	0.22–0.40	0.21–0.41
Confidence interval of specificity	0.081–0.24	− 0.009–0.12	0.21–0.39

### Model interpretation

**Figure 4:**
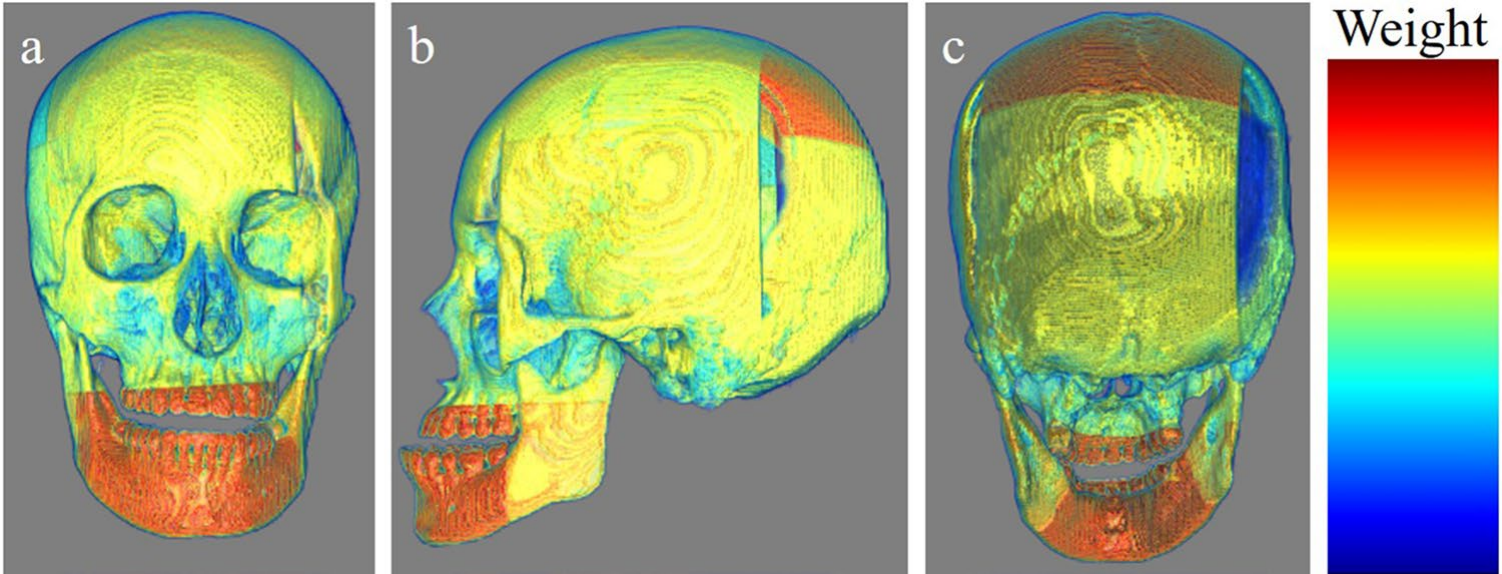
An example that our MIL model predicted appropriately. (**a**) The frontal view. (**b**) The left lateral view. (**c**) The occipital view. The warmer the color, the more weight the patches have.

**Figure 5:**
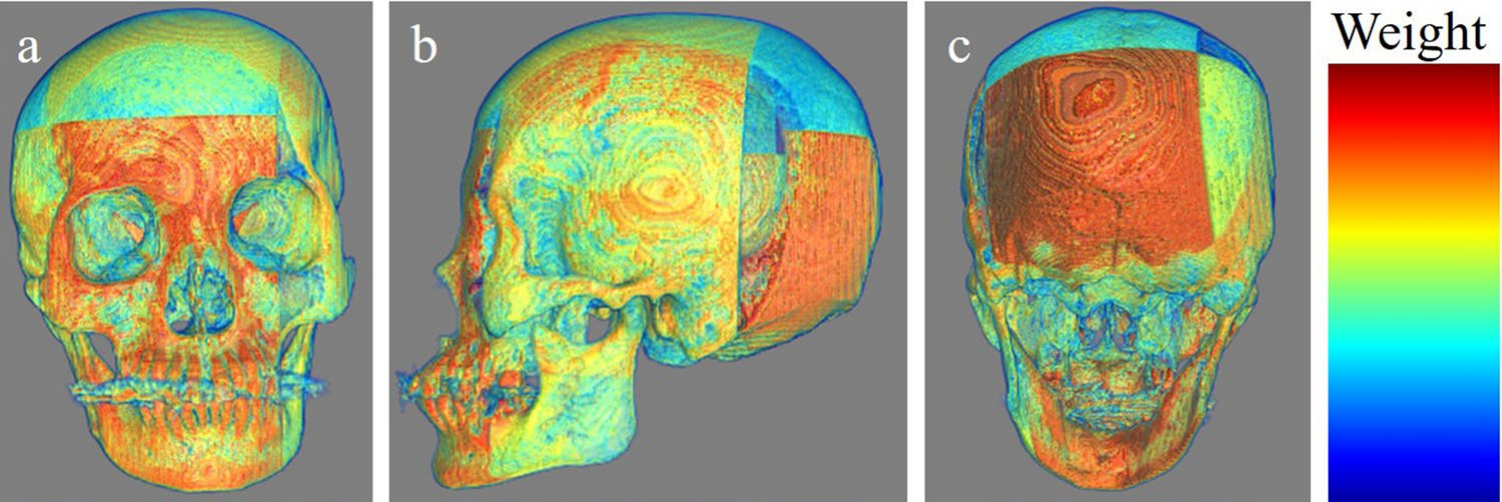
An example that our MIL model predicted appropriately. (**a**) The frontal view. (**b**) The left lateral view. (**c**) The occipital view. The warmer the color, the more weight the patches have.

**Figure 6:**
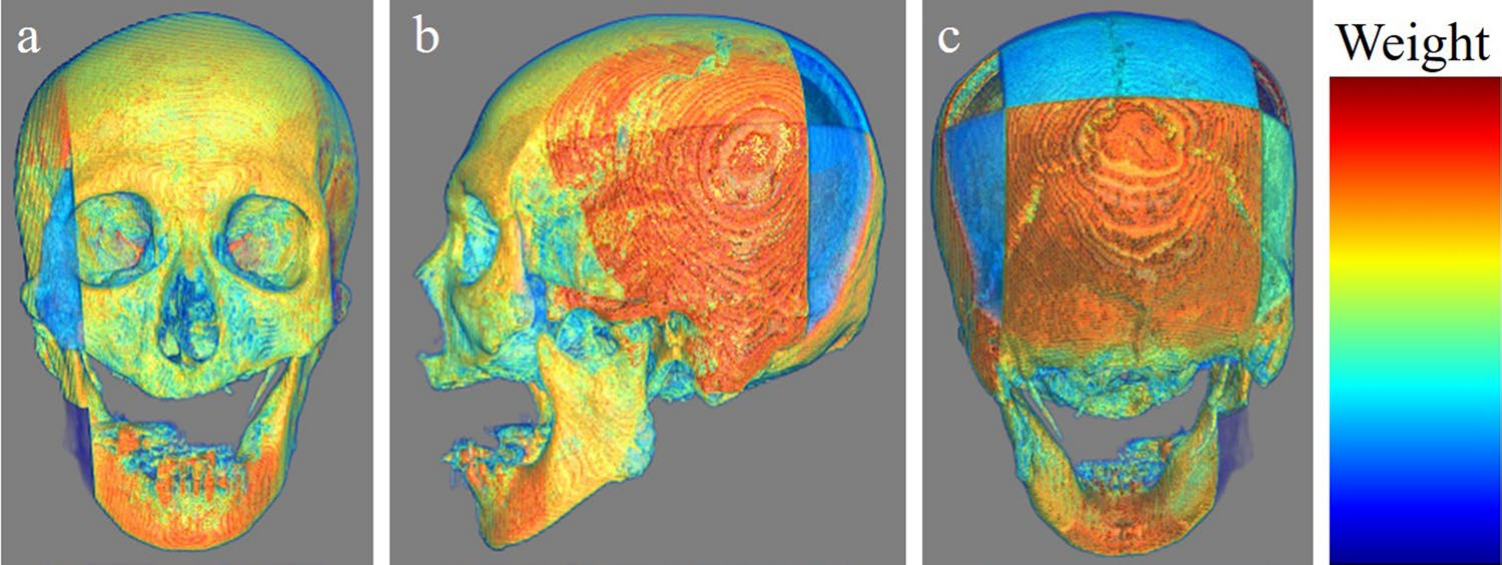
An example that our MIL model predicted appropriately. (**a**) The frontal view. (**b**) The left lateral view. (**c**) The occipital view. The warmer the color, the more weight the patches have.

We depicted model prediction evidence in Figs. 4, 5, 6. These include the cases in which our MIL model correctly predicted sex. The attention-based mechanism can calculate weights for each patch. Therefore, we multiplied the patches by their weights. The color map shows the degree of focus on each patch. Our model seemed to generally focus on the whole skull; however, it focused more around the center of the mandible in these cases.

## Discussion

Our results showed that our model demonstrated higher performance than that of well-trained forensic scientists and absolutely higher performance than that of the beginners, which indicates that the gated attention-based MIL model may be a helpful tool for identifying the sex of cadavers. Our model focused on the whole skull; however, it focused more on the center of the mandible in several cases, as shown in Figs. 4, 5, 6. Mandibles are “U-shaped” in males and “Rounded” in females^16^. Our results suggest that this difference may be critical for sex determination.

Our model incorrectly predicted more females than males. A previous hypothesis suggested that the female skull shape may exhibit masculine characteristics with aging; however, to the best of our knowledge, this hypothesis has never been supported by evidence^17^. In fact, the ages of females who were falsely predicted as males by our model were 81, 83, 74, 65, 55, 90, 84, 84, 48, and 70 years, and most cases were of older age. This theory may complicate sex prediction. Although building models considering age may address this problem, even if we build models that target older cadavers, they would be useless and unrealistic because most forensic cases that forensic scientists must identify lack data regarding personal information including age. The performance of a deep-learning model can generally be improved by increasing the number of training datasets, although our model’s performance surpassed that of humans in this study. The frequency of postmortem CT examinations has recently increased in our country, which indicates that the accuracy of our model can be further improved.

Our model had several advantages. First, it demonstrated reproducibility. Forensic scientists had difficulty identifying the sex in cases where the skull had ambiguous characteristics. For example, reproducibility was limited in cases where the skull had a masculine ridge and a feminine mandible or the skull had characteristics of either sex. In these cases, forensic scientists commonly integrate the sexual features of each area. The final judgment was determined by the decision of the majority because humans could not calculate the probability of each area. We believed that each area had a different probability; therefore, the majority decision-making method may diminish the high probability of one area, leading to identification inaccuracies. In contrast, the attention-based model calculated the probability of each area as a weight, and the final judgment was obtained from these probabilities. This could enhance the interpretability. Second, the performance of the beginner forensic scientist was inferior to that of well-trained forensic scientists. However, this study indicated that individuals with insufficient knowledge and experience may achieve better performance using our model as a supportive tool compared to that by well-trained specialists.

We implemented DenseNet121 from MONAI as a state-of-the-art (SOTA) neural network for feature extraction in MIL; however, MONAI does not provide all the SOTA models. We attempted to build a model with ResNet, a SOTA model provided by MONAI; however, the number of parameters in 3D ResNet was too large for our GPU memory. Therefore, we tried building models with ShuffleNet or SqueezeNet as backbone architectures, instead of DenseNet121. They are resource efficient architectures for 3D CNNs^18^. We referred to their original study to build the models, not MONAI. However, we did not use them as the final model because the performance of Densenet121 for the validation dataset was superior to that of the other two models.

Our model had several limitations. First, our dataset number was relatively small. Generally, deep-learning models require numerous training datasets. However, the definite minimum number of datasets that is needed has not been defined. Several methods can be used to calculate the minimum number of required datasets; however, to the best of our knowledge, there is no optimal calculation method^19^. Rokem et al. demonstrated in their medical image classification that a moderate number of datasets can reach close-to-maximal performance when images are relatively homogenous and the number of effective parameters is sufficiently small^20^. We believe that the images we used were relatively homogenous, and we used DenseNet121, which has a relatively small number of parameters. Therefore, we did not calculate the minimum required number before the training and conducted the training with the available data. However, the range of “relatively homogenous” and “sufficiently small” were vague in the aforementioned study. Therefore, whether our study followed their criteria, “relatively homogenous” and “sufficiently small”, is unknown. However, our model showed better performance than that of the forensic scientists. Besides, MIL is one of the good methods for small datasets. Although we did not think that our data set was generally insufficient based on the above evidence and our results, increasing the number of datasets would further improve the performance of the model. Fine-tuning or transfer learning, which is used to employ well-trained models to other tasks, may also address this problem; however, the number of pre-trained models was small. To the best of our knowledge, there are no models trained with skull images. We considered that models trained with a dataset of images other than skull images, such as brain tumor images, were not applicable to this study. Our main purpose was to build a model with accuracy higher than that of forensic scientists, with reasonable explainability. If we had used pre-trained models with disease datasets like cancer to classify the sex, they might have shown a high performance. However, in our opinion, such models cannot extract essential and important features to classify the sex. Therefore, we did not use pre-trained models. Further, we did not perform k-fold cross-validation though this might have addressed the above problem. Although performing k-fold cross-validation may provide robustness to our model, it would make result interpretation more difficult and challenging. Because both the accuracy and the interpretation of the result were our priority, we did not apply this method. Although less robustness was a limitation, prioritizing the interpretability was our novelty. Second, our model did not focus on the areas that forensic scientists typically focus on in some cases, according to the 3D multiplied weighted images (Figs. 4, 5, 6). For example, forensic scientists typically focus on the muscle ridge of the mandibular angle; however, our model did not do so in the shown cases. We assumed that this may be attributed to patch size. We split whole images into (100,100,100) sizes, with different partial skull volumes in each patch. This patch size, (100,100,100), may have been large. Our model may have assigned more weight to patches containing large volumes, even if they included areas not typically observed by forensic scientists, and less weight to those containing small volumes, even if they included areas typically observed by forensic scientists. We attempted to set smaller patch sizes, such as (30,30,30) or (75,75,75); however, this caused a decrease in accuracy because the patch was too small to capture the characteristics. Developing models that consider the location of each patch, such as TransMIL^21^, may address this problem. However, some skulls for identification may collapse from their anatomical positions, mainly due to fractures. Our training dataset included such cases. Therefore, we assumed that the application of models that consider the location of each patch was not realistic. Finally, our dataset included only cadavers of East Asian ethnicity and the performance of our model for other ethnicities was not determined. In our country, most cadavers to be identified are of East Asian ethnicity; however, identifying the ethnicity of the skull is necessary before applying this model to an unknown skull. Nonetheless, if deep-learning models are trained with data from all ethnicities, this limitation could be resolved. Data obtained from several regions worldwide can be shared to build a more accurate model. Further studies considering these aspects are required.

In this study, a forensic scientist developed all the code. The model developed by a forensic scientist may be inferior in terms of accuracy to the ones developed by specialized machine-learning engineers. Engineers are typically unfamiliar with forensic science; therefore, detailed discussions between forensic scientists and engineers are required to develop machine-learning models. However, this knowledge gap may be difficult to address. Anyone with the minimum knowledge can develop code owing to recent advancements in technology. We considered that model development by forensic scientists may bridge this gap and satisfy the needs of this field. Furthermore, forensic scientists should correctly handle technology as part of their training. Therefore, this study was performed only by forensic scientists.

## Conclusion

Our study showed the utility of deep-learning models for sexual identification in forensic cases. Our model could be a helpful tool for forensic scientists, especially those who have insufficient knowledge and experience in identifying cadaver sex. Tremendous developments have been recently achieved in the field of artificial intelligence, and developments in forensic science by the application of artificial intelligence should be pursued. Our study showed one such development.

## Methods

### Study aims

We aimed to develop a machine learning model with postmortem CT scans and compare its performance with those of forensic scientists to achieve objectivity, reproducibility, and a high level of accuracy in the identification of cadavers. Convolutional Neural Networks (CNN) is a model that uses a deep-learning algorithm to extract image features by multiplying several filters termed “kernels”. CNN mainly comprises four parts: convolutional layers, activation functions, pooling layers, and fully connected layers. In the convolutional layers, kernels, which are small matrices to extract local features, slide over the input image, and element-by-element multiplication and addition are performed. After the convolutional layer, the activation function is applied. This is a nonlinear function that is introduced to learn complex patterns. Next, the pooling layer is applied, wherein the image size is reduced and features are summarized. These manipulations are repeated several times, and general features are obtained. Finally, the output is obtained from the fully connected layer. Owing to this algorithm, the CNN model has good agreement with image cognition^22^.

J Bewes et al.^23^ applied the CNN model to determine the sex from skeletal CT images. In this study, the three-dimensional (3D) Digital Imaging and Communications in Medicine (DICOM) format image was rotated to the left lateral plane using viewers and saved as a two-dimensional jpg format image. We thought that setting the images to the left lateral plane as described in the above report would be easy; however, setting all images to the left lateral plane without any fluctuations was challenging. Thus, the machine-learning model may detect differences in the angle of the left lateral plane, which are caused by human manipulation, as sexual differences between males and females. This model showed excellent performance; however, judgment grounds were unknown in the previous study because the interpretability of the model was not examined. Next, we used 3D images as datasets to develop a CNN model.

### Postmortem CT image datasets

We performed postmortem CT examinations of the cadavers at our institution from February 2022 to April 2023. An Aquilion Helios (CANON MEDICAL SYSTEMS CORP, Tochigi, Japan) CT scanner was used for the examination. The skull was scanned from the mandible to the vertex, with a peak voltage of 120 kV and slice thickness of 0.5 mm. Acquired raw data were reconstructed using the bone algorithm in the DICOM format. We used the bone algorithm to reconstruct images owing to the higher spatial resolution, which reduces the blur caused by the partial volume effect^24^.

### Dataset preprocessing

The datasets contained images of the skull and surrounding tissues, including the cervical spine and brain. We removed the surrounding tissues and extracted only the skull using a workstation (Ziostation2, Ziosoft, Inc., Tokyo, Japan). After this process, the CT image had a size of 512 pixels for the width and height dimensions, whereas the depth varied for each case. This image size was too large for our environment to be applied in CNN model training. Therefore, we resized the images into smaller sizes using TorchIO (version 0.18.90)^25^. TorchIO is a useful open-source Python library utilized to perform data preprocessing, loading, and other tasks. We used TorchIO to change the spatial metadata of DICOM files to 1 mm per pixel in all axes and reorder the orientation to the canonical orientation (right to left, anterior to posterior, superior to inferior). However, the image size of each cadaver varied at this stage. Because deep learning models require all image sizes to be the same, we performed cropping and 0 padding to make all image sizes equal. Then, we could achieve an image size of (300, 300, 300 as depth, height, width), which is smaller than the original image size. Generally, DICOM images contain spatial information, such as pixel spacing, image orientation, and field of view. Through this process with TorchIO, we unified the distance between pixels and the orientation of all images. Next, we binarized the voxels based on their HU values: voxels with HU values ≥ 500 HU were assigned a value of 1.0, and those with HU values < 500 a value of 0.0. HU values commonly depend on the scanning conditions, and the values themselves are meaningless in skull shape evaluation. Generally, a trade-off relationship exists between image noise and spatial resolution in CT image reconstruction, implying that the image noise increases with increasing spatial resolution. Less noisy images have low spatial resolution. We applied the bone reconstruction algorithm as mentioned above, which can present clear spatial resolution; however, the images may become noisy, implying that the dispersion of HU values of the skull is large. We hypothesized that this noise could be reduced using this binarized process because all HU values of voxels of the skull are “1.0”, equally. We decided on a threshold value of 500 HU, according to several studies. Lim Fat et al.^26^ examined the optimal HU values for extracting the cortical bone of the proximal humerus and showed no difference in the 500–900 HU range on bone algorithm-reconstructed CT images. Aamodt et al.^27^ demonstrated the interface between the corticocancellous regions of the femur was indicated by 600 HU. Won et al.^28^ measured the HU values of frontal cancellous bone and showed that the HU value IQR for the group aged < 55 years was 685.0–1022.3 and for the group aged ≥ 55 was 568.1–828.5. To the best of our knowledge, these are the only studies that examined the HU values of cortical and cancellous bones, and none of the previous studies have examined the skull. Nevertheless, we expected that the cortical and cancellous skull bones could be almost completely extracted by setting a threshold value of 500 HU. We chose this threshold because we assumed that certain ranges would be required due to the potential variability of HU values depending on scanning conditions. We confirmed that the skull shape can be clearly extracted by setting this threshold. Finally, we saved preprocessed image datasets in the Neuroimaging Informatics Technology Initiative format, a Medical Image format efficient for image processing and analysis compared to the DICOM format.

**Figure 7:**
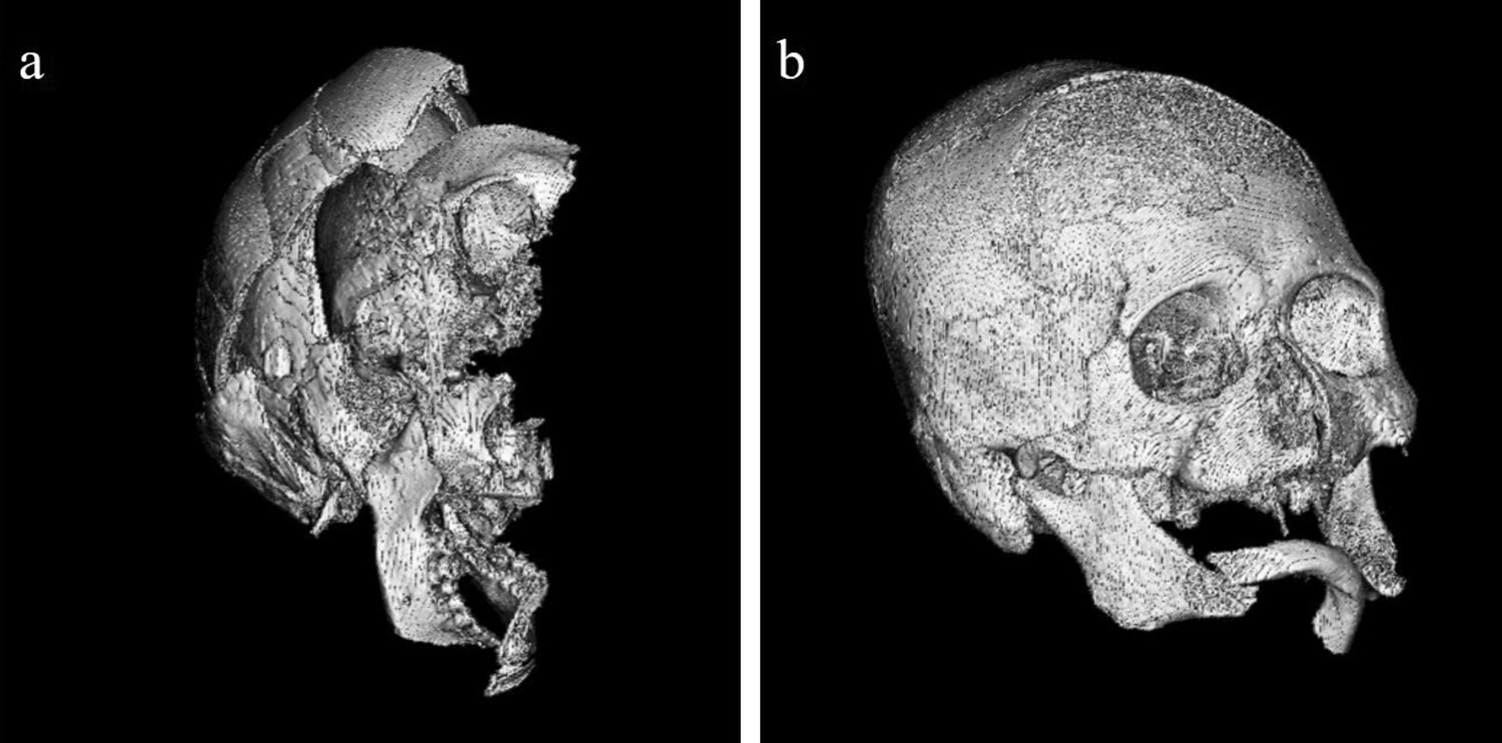
Examples of excluded and included cases. (**a**) This was an excluded case. The skull collapsed, and it was almost impossible to evaluate the sex. (**b**) This was an included case. Fractures of the mandibular bone and right zygomatic bone and burn injury of the parietal bone were confirmed; however, sex evaluation was not impossible.

We established some exclusion criteria during preprocessing: (1) cases aged < 18 years old, (2) cases where forensic scientists could not evaluate the sex due to severe injuries, such as compound fractures or burns (Fig. 7a), and (3) cases that were not from East Asian cadavers. Criteria 2 indicated that we included cases where forensic scientists could evaluate the sex, even with skull injury (Fig. 7b). Criteria 3 was chosen because most cases to be identified are usually of East Asian ethnicity in our country. Generally, the skull shape may differ among ethnicities^29,30^. We could not obtain CT images of cases that were not of East Asian ethnicity; therefore, we anticipated that including a very small number of cases of other ethnicities would affect the performance of the machine-learning model.

### Cadaver age adjustment

We adjusted the age distribution between the male and female groups before initiating machine-learning model training. The skull shape may change with aging^31,32^. Therefore, differences in age distribution between the male and female data may cause the machine learning model to detect features of aging differences, rather than sex differences. We performed propensity score matching for the preprocessed datasets to adjust the age distribution. We used PsmPy (version 0.3.13)^33^, which is a Python library for propensity score matching, with the 1:1 ratio of males to females to balance the data. We applied the K-nearest neighbor as a matching algorithm and set the caliper as 0.2 standard deviations from the propensity score, according to a previous study^34^. Next, we split the whole dataset into three groups, the training dataset, validation dataset, and test dataset, with a ratio of 7:1:2.

### Increasing training datasets by data augmentation

**Figure 8:**
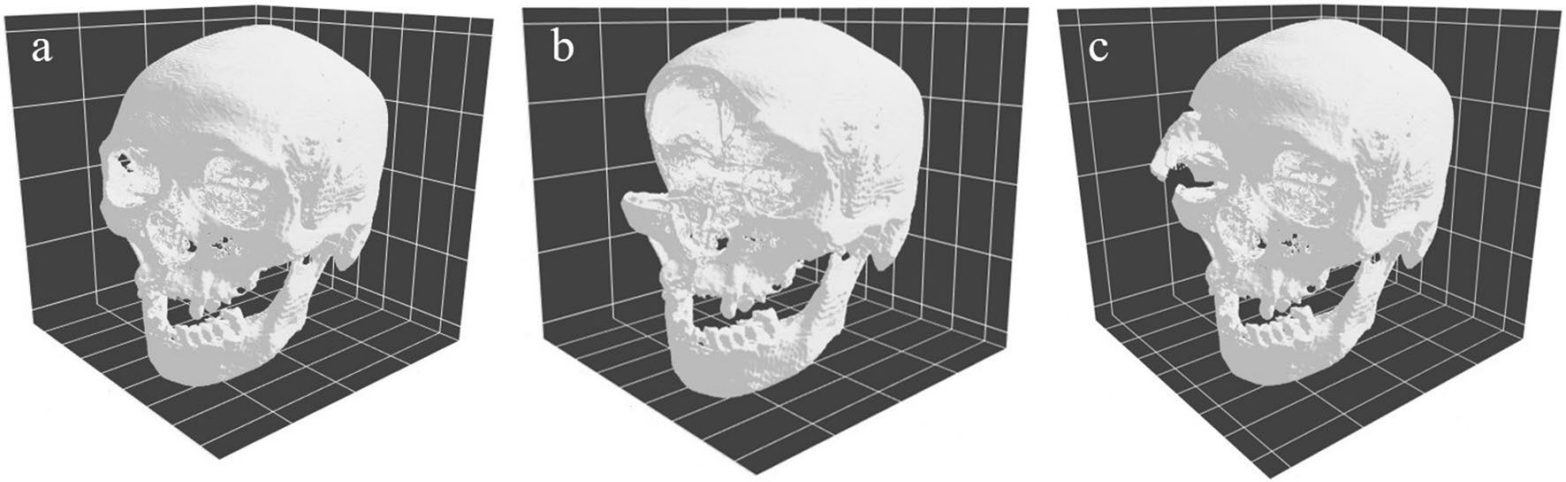
Examples of augmented images. (**a**) This is the original image before augmentation. There is little defection on the left maxillary bone caused by the partial volume effect of the putrefied gas. (**b**) This is the image in which “Cutout” was conducted. The lack of bone around the right orbital can be confirmed. (**c**) This is the image in which “RandomAffinePartial” was conducted. The fracture-like augmentation was generated on the right zygomatic bone.

Data augmentation is a useful technique for deep learning and can artificially increase the number of training datasets by rotating, zooming in or out, or flipping. The augmentation should be performed within realistic limits. TorchIO provides several classes to perform data augmentation on 3D images. We used the “RandomAffine” class for rotating and zooming in or out and the “RandomFlip” class for flipping along the lateral axis. These classes effectively increased the number of datasets; however, forensic scientists may be tasked with identifying the sex with defective or partially injured skulls. We implemented two classes, the “Cutout” and “RandomAffinePartial” classes, to artificially reproduce the above-mentioned situations. The “CutOut” class is a method of randomly deleting a portion of the image. We referred to the original study describing the “Cutout” class^35^. We modified the “RandomAffine” class to implement the “RandomAffinePartial” class. We implemented this class to artificially reproduce the random partial injury of the skull. This class extracted the particular image size from the whole image, applied rotation and translation with the original “RandomAffine” class, and placed it in its original position. The original image and augmented images are shown in Fig. 8.

### 3D-CNN model implementation

Analyzing 3D images requires significant computer resources. We utilized Gated Attention-based MIL to address this issue. MIL is a supervised learning approach^36^. MIL involves the concepts of “bags,” which are labeled, and “instances,” which are unlabeled. A bag comprises several instances and is labeled positive if it contains at least one positive instance. The problem set of MIL is classifying the bags. MIL can be applied to image classification tasks: the images are split into several images of smaller sizes called “patches”, which are used to create a bag. In this case, “patches” means “instances” and a set of “patches” from one image comprises a “bag”. Feature extraction of patches is typically performed using CNN. Finally, the bags are classified. This method reduces computational burden by splitting larger images into smaller ones.

MIL pooling, which is the permutation-invariant function, is used to integrate the instances. The term “permutation-invariant” refers to the output of MIL pooling being independent of the order of the instances. The max-operator or mean-operator MIL pooling methods have been previously suggested. Ilse et al.^37^ believed these methods have disadvantages for model training and proposed the use of the “attention mechanism.” They demonstrated that the attention-based MIL was superior to the existing MIL pooling methods. The attention-based MIL pooling method is defined below:

$$z = {\sum }_{k=1}^{K}{a}_{k}{h}_{k}$$

where:

$${a}_{k} = \frac{exp\left\{{w}^{T} tanh\left(V{h}_{k}^{T}\right)\right\}}{{\sum }_{j=1}^{K}exp\left\{{w}^{T} tanh\left(V{h}_{j}^{T}\right)\right\}},$$

where ${h}_{k}$ is an embedded instance and $w {\in R}^{L \times 1}$ and $V \in {R}^{L \times M}$ are trainable parameters; $z$ is the feature vector that aggregates the representation of all “patches” in a “bag” and is used for classification with multiple layer perceptron. In the formula above, the right side shows the softmax function, which can calculate the weights of each instance. Therefore, the weight ${a}_{k}$ represents the importance of ${h}_{k}.$ Although tanh is a hyperbolic tangent, M Ilse et al. considered that the nonlinearity of tanh(x) is insufficient in the interval − 1–1 for x. This is because the nonlinearity of the activation function usually makes it possible for the model to learn a more complex presentation. Next, they introduced the sigmoid function as “Gate,” as follows:

$$a_{k} = \frac{{exp\left\{ {w^{T} \left( {tanh\left( {Vh_{k}^{T} } \right) \odot sigmoid\left( {Uh_{k}^{T} } \right)} \right)} \right\}}}{{\mathop \sum \nolimits_{j = 1}^{K} exp\left\{ {w^{T} \left( {tanh\left( {Vh_{j}^{T} } \right) \odot sigmoid\left( {Uh_{j}^{T} } \right)} \right)} \right\}}},$$

where $U \in {R}^{L \times M}$ are trainable parameters. The linearity of the hyperbolic tangent was reduced by introducing the sigmoid function. Gated attention-based MIL showed higher accuracy than attention-based MIL in some of their experiments.

The advantages of the attention mechanism in MIL pooling include a smaller number of datasets required for training and improved interpretability. Ilse et al. showed that both MIL models with attention mechanisms can achieve higher accuracy than existing MIL methods, even with small bags or instances. Furthermore, the attention mechanism can set a weight to each instance. This means that evaluators can determine the most important instances. For example, histopathology is the most important factor for the diagnosis of cancer cases, and doctors are often interested in identifying the most important lesions for diagnosis. However, the size of the histopathology whole-slide image is very large. Besides, the number of medical datasets is generally limited. These factors usually impede the training of CNN models. Consequently, Ilse et al. split the histopathology images of the breast and colon cancer datasets into smaller-sized images and visualized these images to understand lesions that were important for cancer diagnosis. On the basis of these results, they believed that attention mechanisms are suitable for medical images. Therefore, we applied the gated attention-based MIL in this study.

**Figure 9:**
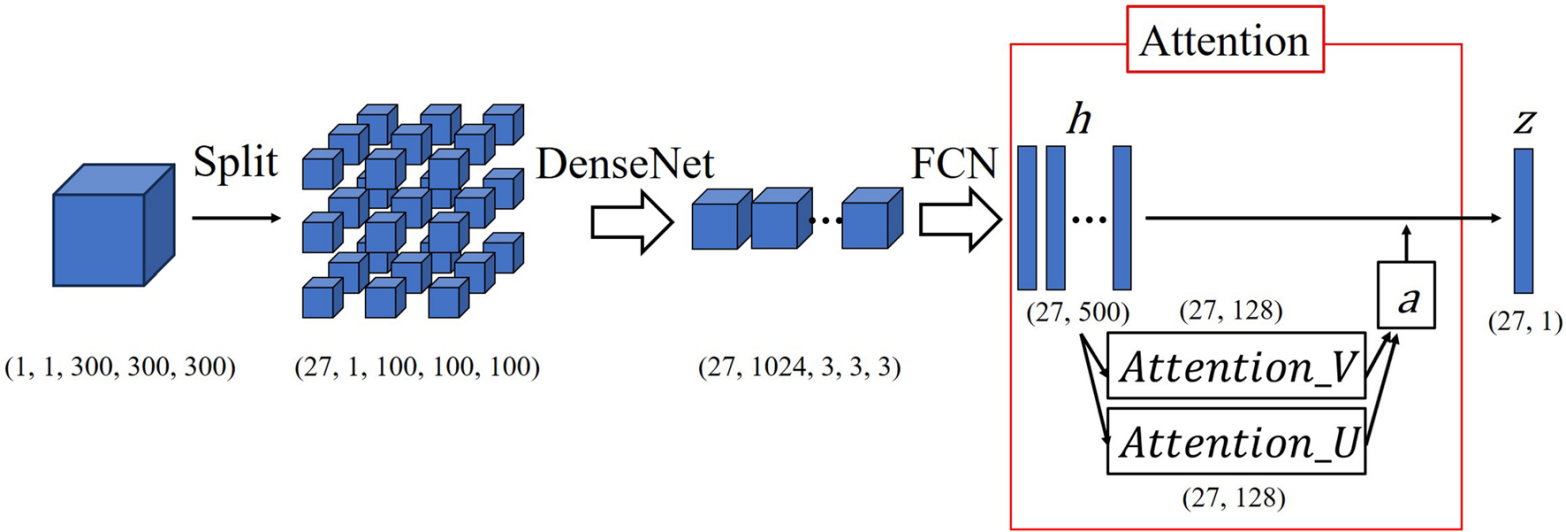
A simple scheme of our model. *h* is an embedded instance, and *z* is a feature vector. Attentin_U is a vector obtained from tanh function, and Attention_V is a vector obtained from sigmoid function. The Hadamard product of these two vectors is used to calculate weights *a*. Finally, z could be obtained by applying the *a* to *h*. *z* is used for classification.

We implemented the codes of Ilse et al.; however, we believed that their feature extraction model, Le-net^38^, was insufficient for our 3D images, based on a few articles that compared the accuracy between Le-net and other models with medical images^39,40^. Therefore, we implemented feature extraction with MONAI (version 1.1.0)^41^. MONAI is an open-source Python library for deep learning in healthcare imaging. To the best of our knowledge, MONAI is the only library that provides several SOTA deep learning models for 3D images. In this study, we applied DenseNet121 from the MONAI library for feature extraction^42^. DenseNet mainly comprises four parts: initial convolution, dense block, transition layer, and classification layer. In the Dense Block, every layer receives the cumulative output of all layers before it. The output of each layer is combined with the input of the next layer. Subsequently, the transition layer is used to reduce the dimension and size. Next, DenseNet will be layered alternately with dense block and transition layer. This mechanism allows the gradient information to be conveyed to the layers, reduces the number of parameters, and facilitates the reuse of feature maps. In summary, we built our model by applying the DenseNet121 as the encoder followed by the Attention mechanism. A simple model scheme is depicted in Fig. 9. The hyperparameters of the Attention mechanism like the number of nodes were the same as in the original paper of Ilse et al. Our model was implemented with PyTorch (version 2.0.1 + cu118) and PyTorch Lightning (version 1.9.3).

The images were divided into a grid, and patches were obtained to perform MIL. TorchIO provides the “GridSampler” class, which can extract patches across a whole volume in a grid. We integrated this class into “Dataloader”, which is essential to train the deep-learning model with PyTorch; grid patches were returned. We set the patch size to (100, 100, 100). This means that our model deals with 27 patches of (100, 100, 100) with channel = 1 because the size of our preprocessed image is (300,300,300).

### Training parameters

We fine-tuned the parameters by executing the training procedure several times. We implemented the “EarlyStopping” class of PyTorch Lightning to stop the training steps when the minimum value of validation loss was not updated more than 50 times in each training procedure. We repeated the training several times and applied the model with the smallest validation loss for the test dataset evaluation. The loss function calculates the discrepancy between the predicted value calculated by the machine-learning model and the actual correct value. We implemented the loss function of Ilse et al. as follows:

$$\text{Negative log likelihood loss} \, = \sum \left\{{Y}_{True}\times log\left({Y}_{Pred}\right) + \left(1-{Y}_{True}\right)\times log\left(1-{Y}_{Pred}\right)\right\}$$

where ${Y}_{True}$ and ${Y}_{Pred}$ indicate the true label and predicted label with the probability, respectively. Machine-learning model training is commonly conducted to minimize the loss value.

### Model evaluation

We used accuracy as the evaluation index. Besides, we also calculated the sensitivity and specificity for females. The following formulas determined accuracy, sensitivity, and specificity:

$$Accuracy = \frac{TP + TN}{TP + FP + FN + TN}$$

$$Sensitivity= \frac{TP}{TP+FN}$$

$$Specificity= \frac{TN}{TN+FP},$$

where TP, True Positive; TN, True Negative; FP, False Positive; and FN, False Negative. We evaluated the performance of the trained model in terms of accuracy, sensitivity, and specificity in the test dataset. Three forensic scientists also identified the sex of the cadavers in the test dataset from 3D images constructed using 3D Slicer (www.slicer.org)^43^. The 3D images were constructed using only skull-extracted DICOM images before preprocessing. Two evaluators were well-trained forensic scientists who passed the Certified medical examination and were certified by the Japanese Society of Legal Medicine. Sex identification is a part of their job profile. The third evaluator was a beginner, with less than 5 years of experience. The accuracy of their results was also calculated. Next, we calculated the 95% confidence interval for the difference in accuracy between the MIL model and each evaluator.

### Training environment

We implemented training and evaluation codes with Python (version 3.9.16) on an NVIDIA GeForce RTX 4090. We used the Windows 11 Pro (version 22H2), CUDA 11.8, and cuDNN 8.9.1 operating systems.

We used EZR (version 1.55, Saitama Medical Center, Jichi Medical University, Saitama, Japan), which is a graphical user interface for R (version 2.7-1, The R Foundation for Statistical Computing, Vienna, Austria), to calculate the 95% confidence interval for the difference in accuracy, sensitivity and specificity^44^.

### Ethics approval and consent to participate

This study was approved by the Institutional Review Board of the Kyoto Prefectural University of Medicine (ERB-C-2749). All experiments were performed in accordance with the Declaration of Helsinki. This study was an observational, retrospective study. The subjects of our research were deceased, and we could not obtain informed consent. Therefore, we followed the Ethical Guidelines for Medical Research Involving Human Subjects, Chapter 5, Part 12–1, (**b**), (ii) (enacted by the Ministry of Health, Labor, and Welfare in Japan). We posted information about our research on our website (https://square.umin.ac.jp/kpum-hoi/research.html), allowing for the exclusion of individuals when their relatives expressed their non-participation in the study. The Institutional Review Board of the Kyoto Prefectural University of Medicine approved this method and waived the need for informed consent.

## Data Availability

The datasets used and/or analyzed during the current study are available from the corresponding author upon reasonable request.
